# Application of virtual simulation technology in sports decision training: a systematic review

**DOI:** 10.3389/fpsyg.2023.1164117

**Published:** 2023-05-18

**Authors:** Ma Yunchao, Ren Mengyao, Li Xingman

**Affiliations:** College of Physical Education and Sports Science, Beijing Normal University, Beijing, China

**Keywords:** virtual simulation, technology, sports, decision training, systematic review

## Abstract

**Introduction:**

Sports decision-making is a complex process and plays a decisive role in sports performance. Virtual simulation technology is one of the popular sports decision making training tools. The application of virtual reality technology in sports decision making training has received widespread attention. The purpose of this study is to evaluate the scientific evidence of the application of virtual simulation technology in sports decision-making training, and summarize its application advantages and limitations.

**Methods:**

The research literature databases of Web of science, PubMed, SCOPUS and Medline were searched, and the results were screened to determine the application of virtual reality technology in motion decision-making. We identified 10 articles and coded them to record the research design, research object characteristics, VR task setting, experimental intervention and research results.

**Results:**

Through the review, it is found that virtual simulation technology has important value for sports decision-making training. In sports practice, virtual simulation technology can simulate sports decision-making tasks, measure and analyze athletes’ sports decision-making performance. We still need to design a more scientific virtual simulation environment for sports decision-making. In this environment, we can better use virtual simulation technology to improve sports decision-making ability.

## 1. Introduction

Decision-making ability is an important perception-cognitive skill in sports, which may play a decisive role in performance. It is defined as the ability to perceive and correctly interpret the information related to the game to select the appropriate sports-specific response. The research on the decision-making process of athletes has been very extensive (Baker et al., 2003). The decision-making level of athletes is the key factor affecting their technical level and sports performance whether in complex collective sports or simple individual ones (Bideau et al., 2009). Decision-making subjects vary in sports decision-making activities, such as a goalkeeper (Hosp et al., 2021), a referee (Gulec et al., 2019; Kittel et al., 2020), and an athlete (Watson et al., 2011). Decision-making tasks, such as intercepting the ball (Hosp et al., 2021), passing the ball (Panchuk et al., 2018), offside (Catteeuw et al., 2010), passing (Watson et al., 2011), and other tasks, provide a reference for the research of various interesting decision-making designs and strategies in sports. However, each mixture of the above variables will produce an interaction that affects the way of motion decision-making.

In recent years, emerging technologies are rapidly applied to sports to improve sports performance and gain competitive advantages (Kittel et al., 2020). Virtual reality (VR) is a technical medium that combines various technologies to improve specific sports performance requirements. It may completely change the field of sports training (Covaci et al., 2012). It is increasingly used in the field of sports and has achieved satisfactory results, especially in sports cognitive training (Zaal and Bootsma, 2011). VR system has the following potential advantages in sports training: standardized scene, enhanced information that guides sports training, and an accurately controlled environment, which can be dynamically changed to create different competitive situations. With these advantages, VR has gained different degrees of success in sports decision-making training, including basketball free throw (Covaci et al., 2012), rugby dodge (Miles et al., 2012; Craig, 2013), handball goalkeeper (Craig, 2013; Cummins and Craig, 2016), football goalkeeper (Miles et al., 2012), football coach (Thatcher et al., 2020), cycling (Driller et al., 2013), and flying ball catch (Silva et al., 2021). Discombe’s research found that cricket batters’ prediction about the landing position is more accurate by using VR training than traditional video with the increase in stimulation correspondence (Discombe et al., 2022).

The main principle of virtual reality technology to improve sports decision-making was to use virtual reality to develop athletes’ perception and cognitive skills and to improve attention, visual observation ability, and sports decision-making ability so as to promote the improvement of sports decision-making ability. More and more evidence shows that cognitive perception training with VR technology can improve athletes’ decision-making ability (Williams et al., 2003; Gabbett et al., 2007). Derek Panchuk’s research shows that using 360 immersive video and head-worn display can improve the decision-making ability of young elite basketball players, which improve their attention and visual observation ability (Panchuk et al., 2018). However, some studies have pointed out that the perceptual-cognitive skill test is based on VR and cannot reliably test the skill differences between dynamic ball players as it lacks the accuracy of the representation of on-field performance (Araújo and Davids, 2016).

The purpose of this study was to summarize the role of VR technology in sports decision-making training with its advantages and disadvantages, to explore its impact on sports decision-making, and to summarize the evidence that it improves the ability of sports decision-making.

## 2. Methods

### 2.1. Participants

Only studies performed on at least one group of competitive and healthy athletes were included. The age, sex, discipline, fitness, or competitive levels of athletes participants were not restricted. A control group was not required. The characteristics of the participants are summarized in Table 1.

**Table 1:** Subject characteristic information.

Study	Country	Subject features				
		Sports	Experience level	Age (years old)	Gender (Male/Female)	*n*
Discombe et al. (2022)	United Kingdom	Cricket	Amateur players	M_age_ = 22.0 years; SD = 4.0 years	14/4	18
Hosp et al. (2021)	Germany	Soccer	Expert, advanced, and novice players	Novice (*n* = 13); 28.64/3.72	NR	33
				Advanced (*n* = 8); 22.00/3.72		
				Expert (*n* = 12); 16.60/1.54		
Kittel et al. (2020)	Australia	Football	Officiated in Division 1 senior Australian football grades in Metropolitan Melbourne competitions.	Mage = 29 ± 13 years	32/0	32
Magnaguagno and Hossner (2020)	Switzerland	Handball	Expert and near-expert handball players	12 expert (M_age_ = 26.17, SD = 3.59)	24/0	24
				12 near-expert (M_age_ = 25.33 years, SD = 4.42)		
Pagé et al. (2019)	Canada	Basketball	Playing at a varsity level	M_age_ = 19.4 years, SD = 3.7 years	21/6	27
Romeas et al. (2019)	Canada	Badminton	University badminton athletes	Mean = 22.98 ± 2.77; (SD) years old	23/6	29
Panchuk et al. (2018)	Australia	Basketball	Elite basketball players	M_age_ = 17.0 ± 0.6 years	10/10	20
Wirth et al. (2018)	Germany	Soccer	15 of them formed the low-skilled group, the other 15 belonged to the high-skilled soccer players	M_age_ = 24.1 ± 3.0 years	30/0	30
van Maarseveen et al. (2018)	Netherland	Soccer	Highly talented female soccer players	M_age_ = 16.3 years, SD = 1.1	0/21	21
Watson et al. (2011)	United Kingdom	Rugby	Non-rugby playing adults	M = 22, SD = 3.1	8/6	14

NR: not reported.

### 2.2. Design and procedures

A literature search was performed on 20 December 2022, using the following databases: PubMed, Web of Science, CNKI, PsycINFO, SPORTDiscus, and IEEE Xplore. For all mentioned databases, the search was conducted using different combinations of the following search terms: [(“virtual reality” OR “Reality, Virtual “OR “Virtual Reality, Educational” OR “Educational Virtual Realities” OR “Educational Virtual Reality” OR “Reality, Educational Virtual” OR “Virtual Realities, Educational” OR “Virtual Reality, Instructional” OR “Instructional Virtual Realities” OR “Instructional Virtual Reality” OR “Realities, Instructional Virtual “OR “Reality, Instructional Virtual” OR “Virtual Realities, Instructional”) AND (“Sport Decision-making” OR “Decision-making Training”)].

A total of 308 articles were selected for initial screening and were used to cover the scope of this review, regarding sports decision-making training, skill, and virtual technology.

Early screening of the articles based on titles and abstracts was performed by two authors (YM and XL). Full-text articles were independently assessed by other authors (YM and XL) (Stang, 2010).

Studies were chosen under the following groups of inclusion criteria: (1) language (only articles written in the English language), (2) publication type (research studies or articles in journals), (3) publication status (only published articles with full-text available), (4) participants (at least one group of subjects were healthy, competitive athletes), (5) type of outcome measures (performance-based, self-reported), (6) intervention of VR (at least one type of VR technology), and (7) sports decision-making (at least one assessment of decision-making skill in sports). The publication search was not time-restricted.

Exclusion criteria were as follows: (1) publication type (conference proceedings are excluded), (2) no detailed experiment design or introduction, and (3) no detailed introduction of the VR environment or the technology used in the study.

### 2.3. Measures

**Figure 1:**
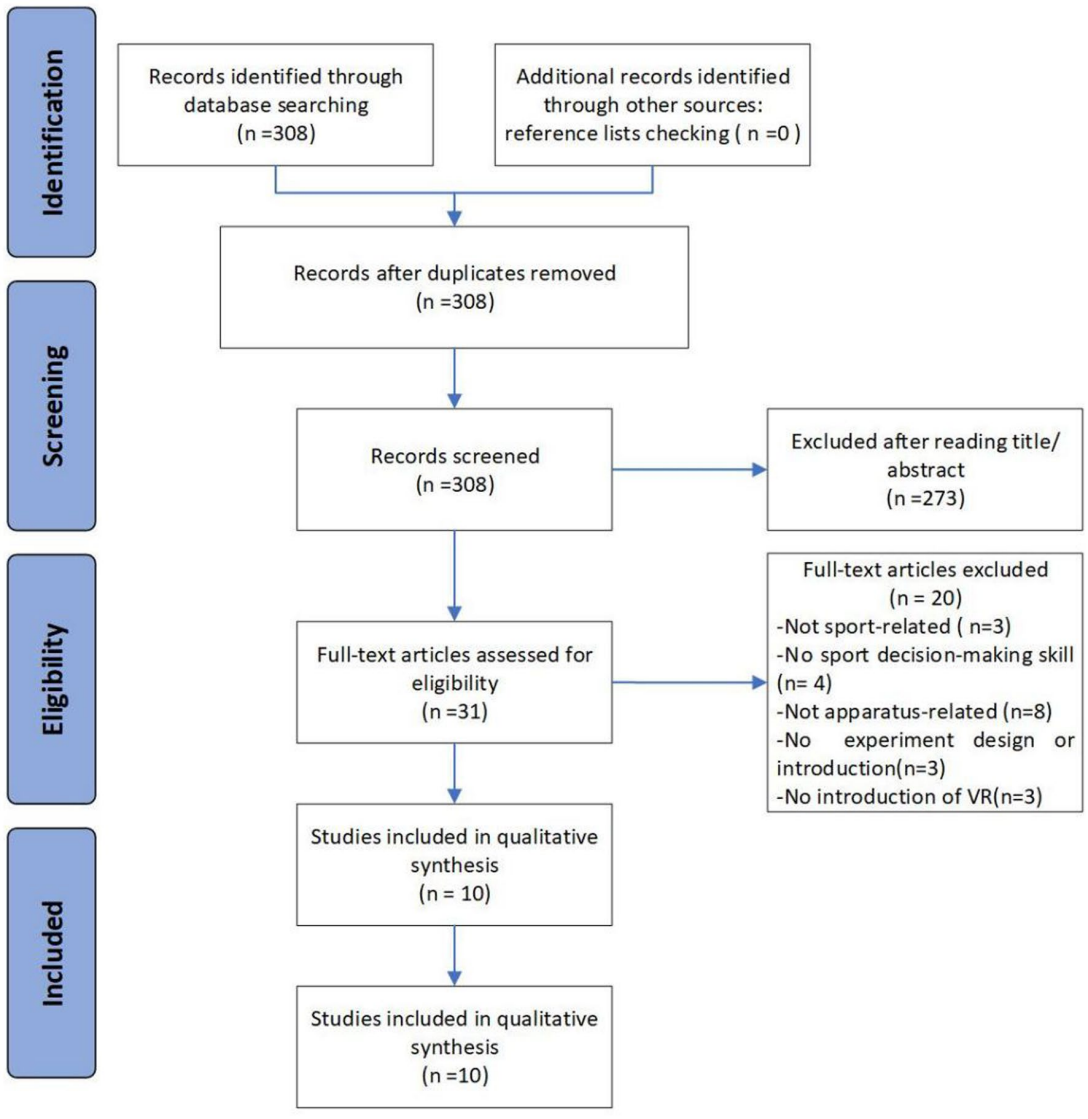
Flow chart illustrating the following steps of the study selection.

Two reviewers extracted the following information independently from the included studies: (1) characteristics of participants (including age, sports discipline, sports level, and training experience), (2) type of VR used (including the creation of the VE and the interactive device), (3) content of sports decision-making (decision-making tasks, decision category, the intervention of VR to decision-making skill, and the outcome of decision-making), and (4) main findings (Figure 1).

The data were collected and are organized in Tables 1–3.

## 3. Results

The purpose of this review was to investigate whether virtual reality training can improve the decision-making ability of athletes (for example, tactical behavior, technical execution, reaction, and decision-making time). Our research results show that the use of virtual reality technology can widely improve the decision-making ability of athletes, and this benefit can be transferred. From the article in the review, it is concluded that the virtual reality environment can help improve the perception and cognitive ability under certain conditions.

This systematic review was screened and included 10 studies, ranging from 2010 to 2022. Most of the studies were published within 5 years and involved a wide range of sports, including football, basketball, badminton, rugby, cricket, and handball. It shows that virtual reality technology has been applied to various sports scenes in the past few years. In the study, it was found that the 3D highly immersive virtual environment has more advantages for the training of decision-making ability than the ordinary 360 video system and the 2D video mode. Immersive virtual reality is mainly used to evaluate decision-making ability and as a training tool to improve decision-making ability, and the research paradigm of experts and novices is commonly used in most intervention research.

### 3.1. Characteristics and level division of participants in different studies

Table 1 shows the characteristics of participants in various studies. According to the review, many studies have strict control over the type of subjects, but in terms of gender, many studies do not use both male and female subjects for experimental research. For example, Kittel et al. (2020) selected 32 male football referees in the study to explore the effectiveness of using 360 immersive video and 2D broadcast video for decision-making improvement. At the same time, the other two experiments on football projects also used single-gender subjects. In the experiment of Magnaguagno and Hossner (2020), who conducted the handball project research, on the influence of the background knowledge of teammates’ defensive quality on the expected performance, only 24 male handball players were used for the whole research. According to the experimental purpose of the research and the characteristics of each project, most of the experimental subjects will generally choose players with the same skill level. For example, in the research on whether video simulation can improve decision-making skills can be transferred and promoted, the subjects are 27 basketball players with the similar competitive level, and so on. However, in some studies, the expert-novice paradigm is also frequently used. For example, when researchers such as Wirth et al. (2018) used the virtual reality 360° video environment to evaluate the cognitive perception of football players, they selected 15 high-skilled and 15 low-skilled football players to experience nine real football scenes from different angles. In addition, Hosp et al. (2021) and others put forward a model based on CNN-BiLSTM to evaluate the specific decision-making tasks of goalkeepers initiating passes in build-up situations. Among the participants, there are 12 high-level expert athletes, eight senior athletes, and 13 novice athletes, to explore how the model classifies the three groups of participants.

### 3.2. Virtual reality environment construction and intervention task setting for different studies

Table 2 shows that different types of virtual reality environments are used in the included literature to prepare for the experimental conditions. Most of the experiments use the scene constructed by 360° virtual reality (360° VR) as the experimental environment or provide video clips for the entire study. Kittel et al. (2020) placed a 360° video camera (360fly 4 k Camera, 360fly, Canons-burg, United States) in a place similar to the referee’s position in the competition to capture the first-person perspective and provide video materials for the experiment. The final video clip presents 360° VR in 4 K video (3,840 × 1,920 pixels). In the same way, Panchuk et al. (2018) also used a 360° camera to shoot the basketball game scene in the experiment of evaluating the effectiveness of immersive video training and played it through the headset display. It is worth mentioning that the virtual reality environment built by Magnaguagno and Hossner (2020) explored the impact of self-generated and acquired background knowledge about the defensive quality of teammates on the expected performance in the complex sensorimotor task. In the experiment, they used five specially arranged GoPro Hero 4 cameras to record at the frequency of 60 Hz, and the camera made the video clip cover at a total angle of 270°. Then, the video editing software (Autopano Video Pro, Kolor SAS, France) was used to import, synchronize, stitch the video clips of each scene, and finally render them into a 360° spherical movie (with valid signals for 270°). The defensive actions recorded by the action capture system (OptiTrack; NaturalPoint Inc., Corvallis, OR, United States) also added real-world stimulation to the experiment.

**Table 2:** VR and task settings in the experiment.

Study	VR and task settings		
	VE (or interactive device)	Decision-making task	Outcome measure
Discombe et al. (2022)	Immersive videos	Verbal responses about landing location prediction and confidence	Prediction accuracy and confidence
Hosp et al. (2021)	360° screenshot of a video presented on the HMD	The responses of participants were relayed verbally with only one right decision available per video	Rated as either right or wrong. The correct decision is a pass to the only teammate who is not covered by an opponent.
Kittel et al. (2020)	All 360°VR footage presented on an HMD	Make decision displayed on the screen	360°VR test performance; Match broadcast test performance
	All match broadcast footage was presented on an iPad		
Magnaguagno and Hossner (2020)	Clips in a life-sized, CAVE environment with projections.	Anticipate whether they should move sideways and tackle the attacker or stay in their position	The detection of patterns, the correctness of motor responses and their positioning to respective teammate’s defensive quality
Romeas et al. (2019)	A fully immersive virtual environment:3D-MOT	Intercept the trajectory of an incoming virtual birdie with a real badminton racket	Dual-task cost; training effectiveness across sessions and between groups
Pagé et al. (2019)	A computer screen/using a virtual reality headset/a computer screen (Head mounted display)	Answer question verbally	Decision making accuracy score
Panchuk et al. (2018)	Immersive video (360°videos in HMD)	Verbalize their decision and were given a ball to simulate their decision	Immersive test performance; SSG performance; Successful performances
Wirth et al. (2018)	Samsung Gear VR headset; (a PS4 controller)	The verbal decision	Interaction concepts and verbal responses
van Maarseveen et al. (2018)	A large screen displayed the video clips	Select what they thought was the best option for the ball carrier	Response accuracy; The gaze behavior
Watson et al. (2011)	Virtual rugby environment with an interactive device (Head mounted display)	Judge the passability of the gap between two virtual defenders *via* a perceptual judgment (button press) task	The accuracy of judgment the passability of the gap between two virtual defenders *via* a perceptual judgment (button press) task

In some team sports, in unpredictable and complex environments, decision-making is limited by time and space, which requires athletes to respond with fast and effective actions (Roca et al., 2013; Gray, 2019; Williams and Jackson, 2019). It is crucial to grasp this feature to improve decision-making skills. For example, in the research of some football and basketball projects, the training task will be divided into decision categories to detect whether the correct judgment can be made within a certain period of time (Pagé et al., 2019). The detection method can be an oral response or the same way as Watson et al. (2011) and others’ research on the perception of the “accessibility” of the gap between two close defenders in the football game, through the perception of judgment (press the button) task, of course, when setting the intervention task. The response time given in the setting of decision-making tasks can also be adjusted according to the accuracy of participants’ decision-making, and the changed content and form will also help improve decision-making skills to varying degrees (Romeas et al., 2019).

### 3.3. Experimental results of different studies in virtual reality environment

In Table 3, the 3D-MOT training method is also worth noting. Romeas et al. (2019) introduced a virtual reality perceptual-cognitive paradigm, which combines three-dimensional multi-target tracking (3D-MOT) with motion or perceptual decision-making tasks. Four different training schemes were used in the experiment, which was conducted in a fully immersive virtual environment. The results show that the dual-task paradigm can achieve satisfactory performance in both tasks. When 3D-MOT is combined with sports tasks, the research results seem to be more conducive to consolidation training in the performance of dual tasks, rather than training at the same time.

**Table 3:** Summary of intervention and experimental conclusions.

Study	Study Design	Intervention	Conclusions
Discombe et al. (2022)	A repeated-measures crossover design	NAN	Cricket batters were able to predict the landing location of the ball with greater accuracy when viewing immersive videos when compared to traditional videos
Hosp et al. (2021)	Model construction	NAN	The expertise recognition model based on eye movements can find valuable features that detect the expertise level of 33 athletes (novice, advanced, and expert) with 73.11% accuracy
Kittel et al. (2020)	A randomized control trial	360°VR group: one video-based training session a week; match broadcast group: completed video-based training session a week; C: did not receive any additional video training	The 360°VR performed significantly better than the control group in the 360°VR retention test. 360°VR was rated significantly higher than match broadcast footage for psychological fidelity, enjoyment, and relevance
			360°VR appears to be a beneficial training tool compared to a control, with stronger engagement from the participants than previously used match broadcast footage
Magnaguagno and Hossner (2020)	Participants took part in the three experimental phases	NAN	Experts are better able to utilize both self-generated as well as explicitly acquired knowledge regarding teammates’ defensive qualities, whereas near experts performance was enhanced only by explicitly provided contextual knowledge
Romeas et al. (2019)	Test the combination between 3D-MOT and a specific motor task’s influence on the training effectiveness	Four training conditions: isolated 3D-MOT task, 3D-MOT combined with a decision-making task, consolidated 3D-MOT and decision-making task, and isolated decision-making task	The dual-task paradigm allowed satisfactory degrees of performance on both tasks despite an important dual-task cost. The results seemed to favor consolidated over simultaneous training for dual-task performance when 3D-MOT was combined with a motor task
Pagé et al. (2019)	Repeated measures on different factors	VR and CS groups: custom-made videos showing from a first-person perspective; CTRL group: watch on a computer screen a video	CS training leads to transferable but non-generalized decision-making gains while VR training leads to transferable and generalized gains
Wirth et al. (2018)	Comparison among groups (high-skilled low-skilled)	NAN	High-skilled had a significantly lower overall recognition-action time
Panchuk et al. (2018)	A training group/the control group	T:10 or 12 immersive video training sessions across 3-weeks; C: in their usual training routine	Benefit of using immersive video for training decision-making skills in team sports
van Maarseveen et al. (2018)	A regression analysis	NAN	The on-field performance of talented soccer players is not predicted by performance on a common set of tests of perceptual-cognitive skill
Watson et al. (2011)	Model construction	NAN	Tau theory can provide a model where the attacker could pick up optical information regarding the difference in the rate of closure of the two gaps, and use this information to inform prospective judgments as to the passability of the gap

Hosp et al. (2021) proposed a model based on CNN-BiLSTM to evaluate the specific decision-making task of goalkeepers passing the ball in a rally situation. They present a scene for each stimulus, which ends after passing it to the user. The experimental results show that this model can detect valuable potential features and detect the professional level of 33 athletes (novice, senior, and expert), with an accuracy of 73.11%. This model is the first step in recognizing the direction of general professional knowledge based on eye movement. In a cricket study, cricket batters have more confidence in their prediction ability when facing people than when facing machines. These results show that the application of immersive video and virtual reality in perceptual-cognitive training may achieve fruitful results. With the increasing popularity of virtual reality technology, sports psychologists should regard this technology as an effective measure to evaluate the expectations of cricket matches.

**Figure 2:**
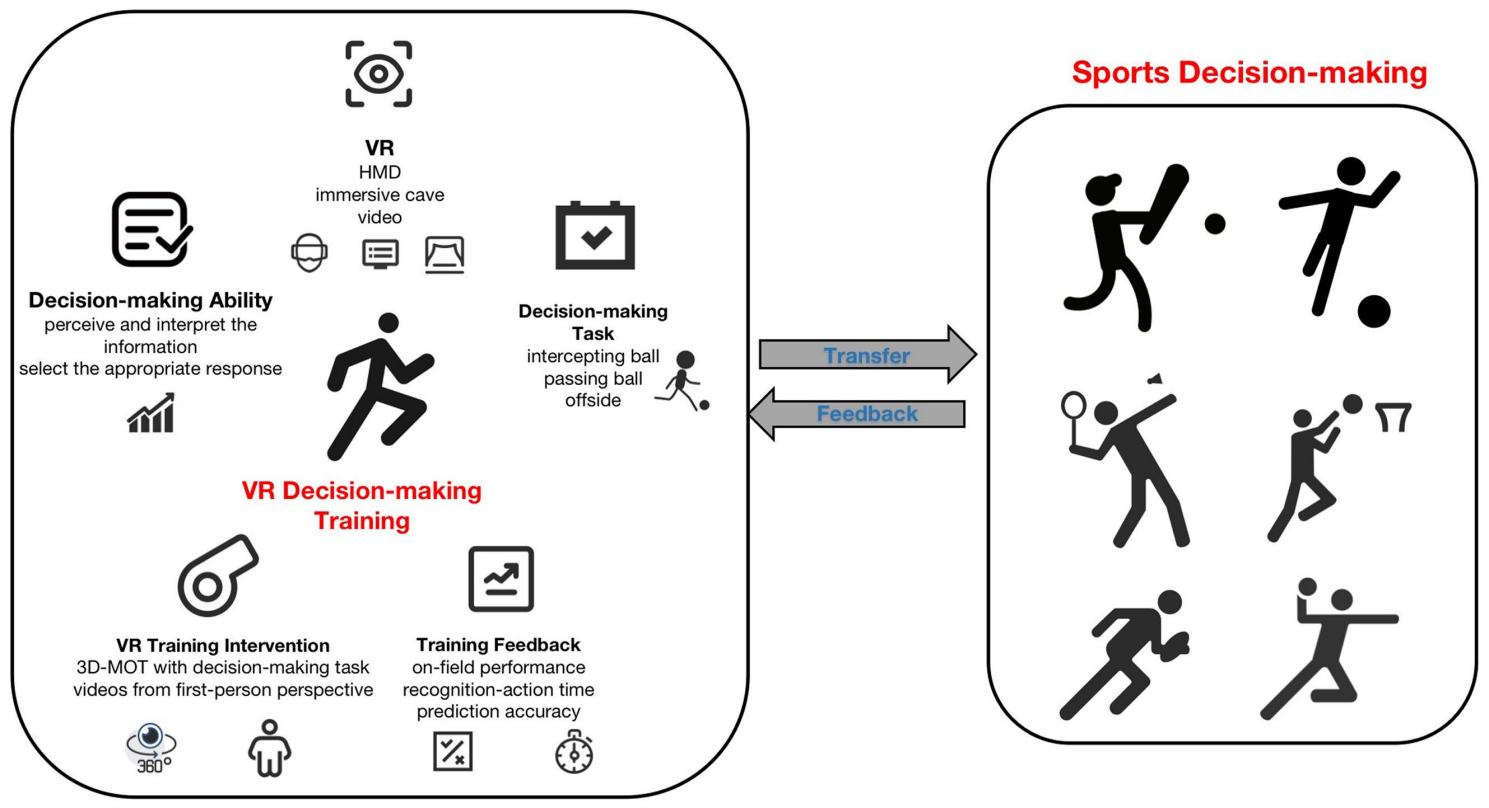
VR decision-making training model.

The model is constructed, which is described in Figure 2, to demonstrate the transfer and feedback between sports decision-making and VR decision-making training. It is concluded from the review that VR is often used as the training condition for the perceptual-cognitive test, but some studies have pointed out that the performance of gifted football players on the field cannot be predicted by a group of common perceptual-cognitive skill tests (van Maarseveen et al., 2018). Some research conclusions also conclude that the virtual reality environment can restore the state of sports practice to a large extent and has a strong ecological validity than other auxiliary training environments. For example, the scores of psychological fidelity, pleasure, and relevance are significantly higher than those of traditional training methods (Kittel et al., 2020) and are also transferable to a certain extent (Pagé et al., 2019).

## 4. Discussion

### 4.1. Reasons why virtual reality is widely used to improve decision-making ability

This review discusses the ways and effectiveness of improving decision-making ability in different projects. In the experiments related to improving decision-making ability, a virtual reality environment with its high ecological effectiveness and psychological fidelity has become the preferred experimental means for most researchers in decision-making training. The essence of virtual simulation technology that can be used to improve decision-making is that it can restore the most real scene state in an unreal environment and to a large extent reproduce the dynamic information source. Then, the decision-making ability is trained and tested by using the decision-making resources included in advance and made. Virtual reality technology enables the variables to be controlled at any time during the whole experiment process and ensures that the peripheral perception, time, and space level of the whole experiment are highly consistent (Liberati et al., 2009). According to the decision-making characteristics, the set experiment requires the participants to use the previous knowledge and experience combined with the complex behavior patterns presented by teammates or opponents under the premise of correct perception of information, making more optimized motion behavior choices (Silva et al., 2020).

The restoration of real decision-making scenes is the most important to improve the ability of real decision-making. Accurate and fast decision-making is the goal of training (Runswick et al., 2018). So in the whole experiment process, different experiments will present short segments of exercise practice in different ways, which will be used by the experimenter to make decision choices and change a variety of decision scenes. After training in “offline” mode, it will become relatively easy.

### 4.2. Main training conditions for sports decision improvement training

There are four kinds of training conditions involved in the research included in this review, namely the traditional 2D video viewing mode, the circular film form, the 360° video recording system from the first perspective, and the highly immersive virtual reality environment presented by some human–computer interaction devices and the fully immersive display Hmd. The purpose of the latter two was to build a virtual environment to present a high-fidelity real experience.

Using the method of constructing a highly immersive virtual environment as the training conditions can not only present a standardized and controlled environment but also reproduce the visual effects in the scene environment, such as the scene atmosphere, audience reaction, and the state of the opponent in the whole game process. At the same time, it can also create a close to real auditory and tactile experience, such as audience cries and the use of specific sensors to make sports equipment reappear in the virtual environment, so the perceptual experience that athletes can perceive during the competition can be reflected in the virtual reality environment.

The research shows that the most widely used training condition is the 360 video system because it can improve decision-making skills to a certain extent (Jung et al., 2021). Compared with building a highly immersive virtual reality environment, the former spends less on equipment and capital requirements and thus is easier to achieve. The immersive video represented by the 360 video system is taken by one of the athletes wearing the 360 video system during the official competition. Then, the participants can participate in the movement from the first perspective by wearing a monitor. Kittel et al. (2020) and others reported that Australian football officials performed better in terms of decision-making accuracy when watching immersive videos than traditional game broadcasting. In addition, immersive video scored higher than traditional video in terms of psychological fidelity, fun, and relevance.

### 4.3. Limitations of VR

Although VR can restore the motion scene to a large extent, there is still some dynamic information that cannot be captured or reproduced. Therefore, because peripheral perception is limited to a certain extent, the user’s vision may be limited. The environment for experience and exploration is slightly different from the real-world scene. However, some scholars (Laufer, 2008) found that limited vision can also block a part of the user’s attention, which also ensures more efficient completion of training tasks. Of course, many early studies supported immersive video because compared with traditional 2D video, the immersive video represented by VR HMD can more realistically replicate the competitive environment because it can stimulate a higher sense of presence, attention, and task participation (Gulec et al., 2019).

Successful performance can be directly attributed to the effectiveness of athletes’ expectations and decision-making ability (Williams and Jackson, 2019). The existence of a virtual reality environment creates more possibilities for the improvement of decision-making ability because it can allow the replication of the same experiment and maintain the judgment “window” of the high time limit (Bideau et al., 2009), as well as the extreme time limit of interceptive sports such as cricket. Athletes need to process information and make decisions in a way that often exceeds the limits of human performance (Cotterill and Discombe, 2016; Runswick et al., 2020). The emergence of virtual reality has slowly transformed us from a dominant way of cognition to perception—the essence of professional knowledge of action and decision-making, that is, the ability to perceive when the environment can withstand certain behaviors and consider our behavioral ability when doing so (Watson et al., 2011).

According to the current research, most of the research finds that the participants make decisions and judgments in the real world while they remain static, so that the persuasiveness of such research results is reduced. At this time, the ecological effectiveness of the participants’ environment is reduced than the actual sports scene (Kittel et al., 2020). Because they only “perceive” rather than “act” when making judgments, their research results are not quite the same when comparing the results of the perceptual task with the results of the perception-action task, so the authenticity of the immersive video is important, but the authenticity of the behavior may be more important for the sports (Craig, 2013). For example, Mann et al. (2010) found that when participants were asked to make actual actions when predicting the direction of cricket, rather than simple verbal reactions, their prediction skills would improve. Therefore, for the form and content of our future training, we should increase the number/type of scenes (Leadbetter and James, 2016). Moreover, the use of scenes with different visual functions (for example, different environments and teams) may be of some help to our research on decision-making skills.

## 5. Conclusion

Through the review, it is found that virtual simulation technology has important value for sports decision-making training. In sports practice, virtual simulation technology can simulate sports decision-making tasks and measure and analyze athletes’ sports decision-making performance. We still need to design a more scientific virtual simulation environment for sports decision-making. In this environment, we can better use virtual simulation technology to improve sports decision-making ability.

The research in this review also gives us some enlightenment. First, when creating immersive content, we should regularly update the decision-making resources to give different incentives to the participants. Second, expert decision-makers can perceive and measure the affordability of actions through visual variables. So, our research should take into consideration the possible impact of visual perception and combine visual perception with other sensory perceptions to study decision-making, for example, add an eye tracker, and consider the relationship between gaze behavior and attention and decision-making, as well as its impact. Finally, as a decision-making evaluation tool, virtual reality has been proven by many studies. If we can set different research contents for novices of experts to comprehensively improve the decision-making ability of various professional levels while increasing the sample size in the research of improving decision-making ability, this will be a big step in decision-making research, and it will also promote the popularization and promotion of virtual reality.

## Data availability statement

The original contributions presented in the study are included in the article/supplementary material; further inquiries can be directed to the corresponding author.

## Author contributions

MY formulated the research goals and aims, developed and designed the methodology, conducted data collection, prepared the published work, specifically writing the initial draft, and acquired the financial support for the project leading to this publication. RM developed and designed the methodology, applied statistical techniques to analyze and synthesize the study data, provided the analysis tool, and prepared the published work, specifically with critical reviews, editing, and revisions. LX conducted data collection, applied statistical techniques to analyze and synthesize study data, and prepared the published work, specifically with critical reviews, editing, and revisions. All authors contributed to the article and approved the submitted version.

## Funding

This work was supported by 2019 Ministry of Education Humanities and Social Sciences Research Youth Project: research on the Construction and Application of Data-driven Intelligent Physical Education Teaching Platform in Colleges and Universities, 19YJC890030 2022 Beijing Social Science Foundation Project: research on the mechanism and path of digital technology enabling high-quality development of public services for Beijing’s national fitness, 22YTB009.

## Conflict of interest

The authors declare that the research was conducted in the absence of any commercial or financial relationships that could be construed as a potential conflict of interest.

## Publisher’s note

All claims expressed in this article are solely those of the authors and do not necessarily represent those of their affiliated organizations, or those of the publisher, the editors and the reviewers. Any product that may be evaluated in this article, or claim that may be made by its manufacturer, is not guaranteed or endorsed by the publisher.
